# Language as a Tool: Motor Proficiency Using a Tool Predicts Individual Linguistic Abilities

**DOI:** 10.3389/fpsyg.2019.01639

**Published:** 2019-07-16

**Authors:** Claudio Brozzoli, Alice C. Roy, Linda H. Lidborg, Martin Lövdén

**Affiliations:** ^1^Integrative Multisensory Perception Action & Cognition Team (ImpAct), Lyon Neuroscience Research Center, INSERM U1028, CNRS U5292, Lyon, France; ^2^University of Lyon, Lyon, France; ^3^Hospices Civils de Lyon, Mouvement et Handicap and Neuro-immersion, Lyon, France; ^4^Aging Research Center, Department of Neurobiology, Care Sciences and Society, Karolinska Institutet and Stockholm University, Stockholm, Sweden; ^5^Dynamique du Langage, Centre National de la Recherche Scientifique, UMR 5596, Lyon, France; ^6^Department of Psychology, Durham University, Durham, United Kingdom

**Keywords:** tool-use, syntax, path model analysis, embodied cognition, language

## Abstract

Different disciplines converge to trace language evolution from motor skills. The human ability to use tools has been advocated as a fundamental step toward the emergence of linguistic processes in the brain. Neuropsychological and neuroimaging research has established that linguistic functions and tool-use are mediated by partially overlapping brain networks. Yet, scholars still theoretically debate whether the relationship between tool-use and language is contingent or functionally relevant, since empirical evidence is critically missing. Here, we measured both linguistic production and tool-use abilities in the same participants, as well as manual and linguistic motor skills. A path analysis ruling out unspecific contributions from manual or linguistic motor skills, showed that motor proficiency using a tool lawfully predicts differences in individual linguistic production. In addition, more complex tool-use reveals stronger association between linguistic production and tool mastery. These findings establish the existence of shared cognitive processes between tool-use and language.

## Introduction

Longstanding theories have linked language and motor skills during development (Greenfield, 1991) and evolution (Darwin, 1871; Hewes, 1973; Rizzolatti and Arbib, 1998; Pulvermüller, 2005). In particular, it has been claimed that complex motor control needed for early advances in human tool-use played a crucial role in the emergence of language (Hopkins et al., 2007; Stout and Chaminade, 2012; Morgan et al., 2015). Tools are mechanical implements that allow to achieve goals otherwise difficult or impossible (Cardinali et al., 2009). The anatomical overlap of linguistic and sensorimotor functions mediating tool-use in the human brain has been extensively documented (Goldenberg and Spatt, 2009; Roby-Brami et al., 2012). The motor-cognitive complexity necessary for the use of tools (Vaesen, 2012), relies on structures lateralized to the left hemisphere, in parietal (Supramarginal gyrus, SMG) and premotor cortices (Inferior Frontal Gyrus, IFG; Lewis, 2006; Higuchi et al., 2009; Valyear et al., 2012; Gallivan et al., 2013; Peeters et al., 2013). The involvement of those regions in linguistic tasks has been shown both in brain-damaged patients (Buxbaum, 2001; Iacoboni and Wilson, 2006; Fazio et al., 2009) and healthy individuals (Embick et al., 2000; Pa and Hickok, 2008). Furthermore, functional activations related to tool-use and language understanding overlap within this network (Higuchi et al., 2009). Recent behavioral data have highlighted similar perceptual changes after use of a tool or language in order to enter in possession of an object, showing possible link between those two domains (Scorolli et al., 2016). Yet, whether the anatomical co-localization of tool-use and language related activations is just contingent or also functionally relevant is still matter of debate. This is mainly because behavioral evidence is critically missing of a link between the ability to use a tool and to produce language.

**FIGURE 1:**
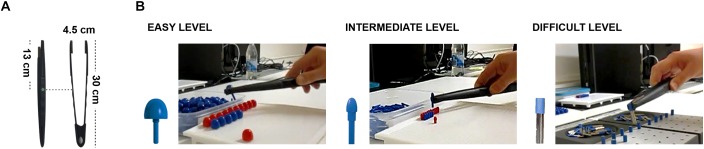
Experimental Methods and Results. **(A)** The tool employed. **(B)** The motor task: big, small, and grooved pegs for the easy, intermediate, and difficult level, respectively (see Supplementary Videos). Participants performed all tasks both with the tool and the hand, in separate blocks.

Here, we aim to show that tool-use and language share cognitive processes. In order to test our prediction, we measured both linguistic production abilities and tool-use abilities in the same participants (*N* = 32, right-handed native Swedish speakers, age mean ± SD: 25 ± 4 years old). We tested the specificity of the link between tool-use and language by controlling for general motor skills. As control for tool-use, we measured participants’ manual motor skills, asking them to solve the same motor task with the bare hand (Figure 1). As control for linguistic production abilities, we assessed participants linguistic motor skills via a Sentence Repetition task where a similar motor and phonological, but not linguistic elaboration of verbal material was required. With a path model controlling for the explanatory contribution of manual and linguistic motor skills, we revealed that tool-use proficiency predicts individual linguistic abilities. Furthermore, by systematically varying the complexity of the action with the tool, we could show that the most complex action displays the strongest association between linguistic production and tool-use performance. Our findings show that tool-use and language production are behaviourally linked crucially supporting the hypothesis that the anatomical overlap of tool-use and linguistic functional activations might correspond to common cognitive processes. This evidence supports the evolutionary hypothesis that tool-use behavior might have played a crucial role for the emergence of cognitive functions necessary for language.

## Results

### Linguistic Performance

**FIGURE 2:**
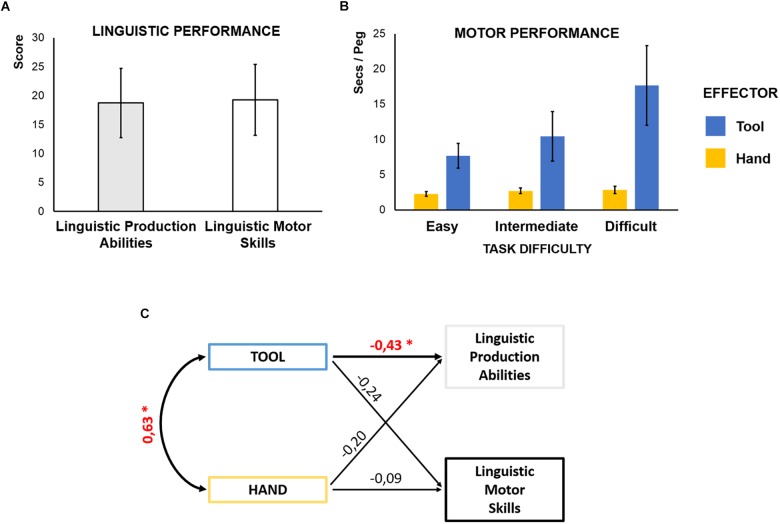
**(A)** Linguistic performance: mean and standard deviation (SD) of the scores in linguistic production abilities (gray bar) and linguistic motor skills assessments (white bar; mean ± SD: 18.8 ± 6.0 in the linguistic production and 19.3 ± 6.1 in the linguistic motor skills scale; 2-tailed *t*-test, *t* = 0.50, *p* = 0.63). **(B)** Motor performance: mean and SD of the motor skill (seconds/peg) when the task was performed with the tool (blue bars) and the hand (yellow bars), across different levels of complexity. For both effectors, the time to correctly place a peg increases with the complexity of the task (Main Effect of Complexity): For the hand [*F*(2,62) = 57.2, *p* < 0.001, η^2^ = 0.65]: mean ± SD (secs/peg) 2.29 ± 0.33 in the easy level, 2.72 ± 0.40 in the intermediate level, and 2.85 ± 0.52 in the difficult level; all Newman–Keuls *post hoc* tests *p* < 0.05. For the tool [*F*(2,62) = 73.4, *p* < 0.001, η^2^ = 0.70]: mean ± SD (secs/peg) 7.69 ± 1.74 in the easy level, 10.46 ± 3.52 in the intermediate level, and 17.66 ± 5.65 in the difficult level; all Newman–Keuls *post hoc* tests *p* < 0.01. **(C)** Shared processes between language and tool-use: Path Model testing the prediction of an association between motor performance with the tool and individual linguistic abilities, by controlling for the explanatory contribution of both manual and linguistic motor skills. The figure reports the standardized estimates for each prediction. Crucially, tool-use performance is a significant estimate of individual linguistic production abilities (see Table 1). ^∗^*p* < 0.05.

We assessed individual linguistic production abilities with the “Sentence Construction” scale of the BeSS battery designed to detect subtle language disorders in Swedish (Laakso et al., 2000; Berg et al., 2003). At each item, the task requires to semantically and syntactically process three or four words and produce a correct sentence featuring all of them in the given syntactic form. To control for general motor resources also engaged to verbalize linguistic material, we measured linguistic motor skills with the “Sentences Repetition” scale of the BeSS. At each item, participants are requested to read and repeat a sentence aloud. Importantly, in this task participants can use semantic and syntactic contents for retrieval but, contrary to the previous assessment, no further processing of verbal material is required to articulate the already well-formed sentences. Two independent judges rated participants’ performance in each scale. Reliability between raters, measured by intraclass correlations (ICCs), was high (0.84, 95% Confident Interval (CI): 0.70 – 0.92 for linguistic production abilities, and 0.92, CI: 0.96 – 0.99 for linguistic motor skills). The average scores in the two assessments were comparable (Figure 2A) and the correlation between the two scores was not significant (Pearson’s *r* = 0.27; *p* = 0.14).

### Motor Performance

We measured participants’ ability to use a tool while playing peg-board games. Participants were instructed to correctly grasp and place as many pegs as quickly as possible by using a 30-cm long mechanical pinch with their right hand (Supplementary Videos). In separate blocks, participants faced three levels of complexity, which required to deploy progressively more complex sensorimotor skills. *Easy* and *intermediate* levels differed for the size of the circular pegs employed (big and small, respectively); in the *difficult* level, pegs were grooved requiring to embed an additional rotational component in the grasp-and-place action (Figure 1B), resulting in a more complex hierarchy to be handled among the motor sub-units of the action: grasp-*rotate*-and-place. We measured the number of correctly placed pegs and the time necessary for that, in each block. We expressed the tool-use ability as the average time per correctly inserted peg (seconds/peg). To disentangle skills specific to tool-use abilities from those related to general manual motor skills, we asked them to perform the same task (including the same three levels of complexity) with the bare right hand. Two ANOVAs run on the performance with the two effectors separately, with Complexity as factor, revealed that the time required to correctly place a peg increases with the complexity of the task (Figure 2B).

### Tool-Use and Linguistic Abilities Share Cognitive Processes

For effective tool mastery, the motor system needs to handle increased complexity (Stout and Chaminade, 2012). In our prediction, the same cognitive functions carry out these tasks not only in the motor but also in the linguistic domain. We therefore expect that better tool-use is associated with better linguistic production performance, independently of individual manual and linguistic motor skills. We defined a path model with tool-use performance as predictor of Sentence Construction scores. We controlled for manual and linguistic motor skills entering them in the model as independent predictors of both tool-use and linguistic production abilities (Figure 2C). The model therefore tests the crucial hypothesis that individual tool-use performance predicts linguistic production abilities, independently of individual manual or linguistic motor skills [χ^2^(1, *n* = 32) = 0.42; *p* = 0.52; RMSEA < 0.001, pclose = 0.53; NFI = 0.99; Table 1]. As expected, manual motor skills are significantly correlated with the motor performance with the tool (st. β = 0.63, *p* = 0.003). More critically, individual motor proficiency using the tool provides a consistent estimate of linguistic production abilities (st. β = -0.43, *p* = 0.02): better tool-use (less time needed to correctly place a peg) predicts better linguistic production. Crucially, the performance with the hand without the tool does not significantly (contribute to) predict linguistic production abilities (st. β = -0.20, *p* = 0.28). Furthermore, tool-use performance does not significantly predict linguistic motor skills (st. β = -0.24, *p* = 0.28), neither do manual motor skills (st. β = -0.09, *p* = 0.67). This allows to rule out that general motor skills might explain the shared variance between tool-use and linguistic production abilities. We tested alternative models by constraining the non-significant paths between tool-use performance and linguistic motor skills [χ^2^(2, *n* = 32) = 1.58; *p* = 0.45; RMSEA < 0.001, pclose = 0.48; NFI = 0.95] or the path between manual performance and linguistic production abilities [χ^2^(2, *n* = 32) = 1.56; *p* = 0.46; RMSEA < 0.001, pclose = 0.49; NFI = 0.95]. However, the original model provided the best data fitting. This findings reveal shared cognitive mechanisms for tool-use and language production, after controlling for the explanatory contribution of manual and linguistic motor skills. This strongly suggests that the anatomical overlap of functional activations related to tool-use and language have a direct behavioral significance and should be viewed as common anatomo-functional resources.

**Table 1:** Path model, standardized and unstandardized estimates of the model per each prediction and relative standard error (SE) and significant level.

	St. Estimates	Unst. Estimates	S.E.	*p*-value
HAND ↔ TOOL	0.63	0.77	0.26	<0.01ˆ*
TOOL → LPA	-0.43	-0.72	0.32	0.02ˆ*
TOOL → LMS	-0.24	-0.44	0.41	0.28
HAND → LPA	-0.20	-2.95	2.74	0.28

*LPA, Linguistic production abilities; LMS, Linguistic motor skills.*

### Increased Tool-Use Complexity Shows the Strongest Association With Linguistic Abilities

**FIGURE 3:**
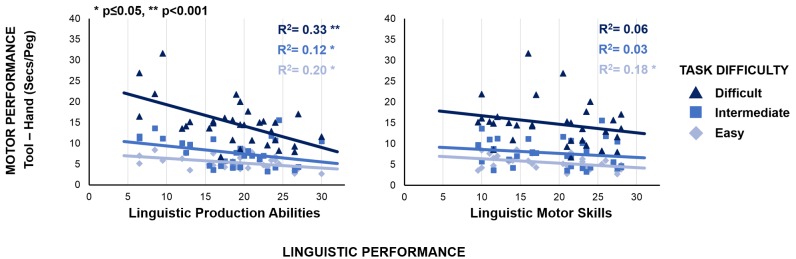
Link between linguistic production abilities and tool-use performance: the association between motor performance and linguistic production abilities increases with the complexity of the motor task (**left panel**; *y* = –0.1^∗^*X*+7.6, *R*^2^ = 0.20, and *p* < 0.05 in the easy task, light blue diamond markers and line; *y* = –0.2^∗^*X*+11.3, *R*^2^ = 0.12, and *p* = 0.05 in the intermediate task, blue square markers and line; *y* = –0.5^∗^*X*+24.5, *R*^2^ = 0.33, and *p* < 0.001 in the difficult task, dark blue triangle markers and line). Importantly, this functional relationship is absent between linguistic motor skills and tool-use performance (**right panel**; *y* = –0.1^∗^*X*+7.5, R^2^ = 0.18, and *p* < 0.05 in the easy task; *y* = –0.1^∗^*X*+9.6, R^2^ = 0.03, and *p* = 0.33 in the intermediate task; *y* = –0.2^∗^*X*+18.8, *R*^2^ = 0.06, and *p* = 0.19 in the difficult task).

Additional findings show that tool-language relationship is modulated by tool-use complexity, which we systematically varied. We expressed individual aptitude in tool-use by calculating the time (i.e., the cost) required to complete the task with the tool as compared to the hand, within each complexity level. This measure is calculated individually as the difference between the time employed to complete the task with the tool and the time employed to complete the task with the bare hand, in each condition. It reflects the ability to cope with the motor constraints introduced by the tool, by controlling for manual motor skills: more skillful tool-use corresponds to lower cost. If shared processes between tool-use and linguistic production abilities reflect the mastery of complex hierarchies, the cost should co-vary with the linguistic production performance. The most complex motor task (grasp-rotate-and-place) should show the strongest association between tool-use cost and linguistic production scores. An ANOVA with Complexity as categorical, and both Sentence Construction and Repetition scores as continuous factors confirmed this prediction, by revealing a significant interaction between Complexity and Sentence Construction score [*F*(2,58) = 3.4, *p* = 0.04, η^2^ = 0.10]: the most complex motor task shows the strongest association between linguistic production abilities and tool-use performance (Figure 3, left panel). This functional relationship is absent between linguistic motor skills and tool performance [Figure 3, right panel; *F*(2,58) = 0.1, *p* = 0.95].

## Discussion

These findings provide unprecedented empirical evidence of common cognitive mechanisms for tool-use and language production. The link between tool-use and linguistic production abilities indicates that the partial overlap of the two functions onto a similar network as indicated by distinct neuroimaging studies on tool-use or linguistic processes, is not contingent but has important behavioral relevance. In particular, our path model shows that tool-use and linguistic production share cognitive processes. Both language production and skillful tool-use might need a contribution of executive functions. According to this account, the shared variance between linguistic production and tool-use abilities highlighted by the path model analysis, might be explained by participants’ executive functions. For instance, previous research showed the contribution of working memory for language processing (Makuuchi et al., 2009). Similarly, effective tool-use might rely on a solid working memory capacity, as suggested by data on brain-damaged patients presenting symptoms of dysexecutive syndrome (Goldenberg et al., 2007). The Sentence Repetition task we employed as control for motor and articulatory skills also involves an important working memory component. The hypothesis that the shared variance between tool-use and linguistic production might be due to working memory abilities deployed for both tasks, predicts a significant link between tool-use performance and the score in the Sentence Repetition task. However, such a path is non-significant in our model. This might be due to statistical power limitations of our study or suggest that the shared functional core between linguistic production and tool-use might reflect the contribution of cognitive functions other than executive functions.

According to an alternative account, indeed, language production and tool-use might share a form of syntax, a cognitive function, distinct from working memory and dedicated to handle complex structures of elements (Makuuchi et al., 2009). In support of this account, findings during the last decade suggest that the ability to process syntactic and recursive structures is not exclusive to language but might extend to the motor domain (Fadiga et al., 2006; Pulvermüller, 2014). Actions, indeed, consist of components (e.g., reaching, rotation, grip) which are not independent and need appropriate ordering (Koechlin and Jubault, 2006; Roy et al., 2013). Accordingly, syntactic processes have been shown to handle complex structures irrespectively of the fact that the cognitive material is made of linguistic or motor elements (Roy and Arbib, 2005; Fazio et al., 2009; Clerget et al., 2012; Alamia et al., 2016). This view is supported by our result showing that linguistic production abilities covary with a measure of tool dexterity. Such a covariation appears to be stronger when participants have to solve the most complex actions with the tool. This can be interpreted as the fact that shared processes between motor and linguistic production abilities reflect the mastery of complex hierarchies. At the easiest level of the motor task, a significant correlation exists between the motor cost in using the tool with respect to using the hand and both the scores in the Sentence Construction and the Sentence Repetition tasks. Crucially, however, the performance in the most complex motor task (retrieve-rotate-and-fit), therein requiring more “motor syntax” compared to the two other levels of difficulty, displays the strongest selective association with linguistic production abilities, but not with the linguistic motor skills. These results together suggest that the easiest level of the motor task is not the most optimal one for highlighting the specific relationship between tool-use and linguistic production abilities. The differential effect in the relationship between tool-use performance and linguistic production abilities rather than with linguistic motor skills, appears at the most complex level of tool-use. In this condition, the action requires to embed an additional component within the motor plan, which might represent a more complex motor syntax. At this regard, neuroimaging studies on tool-use have pointed to a role of the Basal Ganglia structures in the temporal segmentation and sequencing of motor acts during tool-use (Obayashi et al., 2001). On the other hand, Basal Ganglia play an important role in syntactic processes (Ullman, 2006). We suggest that Basal Ganglia might be a crucial node of the shared network between tool-use and language.

In an evolutionary perspective, recent studies on great-apes have provided evidence of an association between neuroanatomical asymmetries in correspondence with SMG and IFG, and handedness for tool-use (Hopkins et al., 2007; Gilissen and Hopkins, 2013; Meguerditchian et al., 2013) as well as for manual gestures (Hopkins et al., 2005). These results suggest that tool-use may have served as a preadaptation (Hopkins et al., 2015) for the emergence of motor and cognitive functions associated with communication. Compatibly, archeological findings estimated that early hominids started to extensively use tools approximately 2.5 million years ago (Roche et al., 1999), before the appearance of language (d’Errico et al., 2003). Our results support the theory of a common evolutionary origin for cognitive functions underlying tool-use and language (Anderson, 2010; Stout and Chaminade, 2012): increasingly effective tool-use exerted selective pressure for the emergence of cognitive resources able to process complex hierarchies in the motor domain (Stout and Chaminade, 2012; Uomini and Meyer, 2013). This new functionality was then exapted for linguistic purposes (Anderson, 2010).

## Conclusion

In conclusion, our results highlight the existence of shared cognitive processes between tool-use and linguistic production. These novel findings provide behavioral support for the hypothesis that the anatomical overlap of tool-use and language related activations reflects a functional link between these domains. We suggest that common syntactic processes exist for the two domains.

## Materials and Methods

### Participants

Thirty-two participants (15 female; age mean ± SD: 25 ± 4 years old), recruited through ads in the university, websites and local newspapers, took part in the study for compensation (100 SEK). All participants were right-handed (Oldfield, 1971; score mean ± SD: 8.2 ± 1.9), native Swedish speakers, had normal or corrected-to-normal sight, and did not report any motor nor neurological problems. All the procedures employed have been approved by the ethics committee (Regionala Etiksprövningsnämnden of Stockholm) and participants gave their written informed consent.

### Tasks

Linguistic and motor performance have been measured, in this order, in each participant. Linguistic performance was audio-recorded (Olympus Digital Voice Recorder, WS-833; sampling frequency 44.1 kHz). Motor performance was video-recorded (Canon IXUS145, HD-videos 1270×640, sampling frequency 25Hz).

#### Linguistics Assessments

We measured linguistic motor skills and linguistic production abilities by employing the “Sentence Repetition” and “Sentence Construction” scales, respectively, part of the test battery BeSS (Bedömning av subtila språkstörningar, Assessment of subtle language disorders). This battery was developed at the Department of Logopedics and Phoniatrics of Göteborg University in Sweden and is meant to detect subtle linguistic problems in adults. In particular, it has already been employed to assess linguistic abilities in patients presenting motor deficits, with the aim to show a correlation between the linguistic and the motor behavior (Laakso et al., 2000; Berg et al., 2003). In BeSS, the performance is judged item by item using a three step scale: 0, 1, or 3 points. Two independent judges rated each participant’s performance. The two tasks involve linguistic production, in order to have a motor output in both linguistic behavior and, obviously, in tool-use. This ensured more homogeneity across our tasks.

##### Sentence repetition

Participants were presented with a list of written sentences in front of them. At each item, participants were instructed to read a sentence, and then repeat it aloud word by word exactly. Participants were not allowed to look at the sentence while repeating it. The experimenter checked that participants were complying with the instruction. The test consisted of one practice trial and ten items. To get a full score, the participant needed to read and repeat the sentence exactly as given, without changing the word order. The maximum score in the scale was 30. The length of the ten sentences designed to be included in the test spans from 9 to 16 words, from 15 to 24 syllables. The complexity of the sentences is such that they could be found in a daily newspaper or a contemporary novel and the words have similar frequency of occurrence in Swedish language. The task gives a measure of participants’ ability to verbally repeat a list of words organized in a correct sentence. As such, the task gives a measure of linguistic motor skills and phonological memory necessary to verbalize a sentence, without requiring any further syntactic or semantic elaboration. The following criteria were used for rating:

(a) Misplacement of words, use of synonyms and any change or mistake in the reading material counted as a reduction of 1 point;
(b) The omission of a significant chunk of the sentence in the repetition resulted in a 0 score to the item.

##### Sentence construction

At each trial, participants listened to the indication of a context followed by a list of three or four words, each with a specific syntactic form. Participants had to construct one sentence including all of them. The test included one practice trial and ten items. To receive a maximum score, the sentence produced by a participant had to feature all of the words, in the given syntactic form and to be correct and sound. The maximum score in the scale was 30. The following criteria were used for rating:

(a) Initial loud repetition of the given words not organized in a sentence resulted in a reduction of 1 point;
(b) The use of one of the words in a different syntactic form with respect to the given one resulted in a reduction of 1 point;
(c) The absence of one of the given words in the response was rated as 0 points;
(d) A long latency of response (more than 20 s for the first word) or a long pause in the production of the sentence (10 s between two words) resulted in a reduction of 1 point;
(e) Unusual but correct constructions or sentences carrying unsound meaning resulted in a reduction of 1 point;
(f) Sentences with incorrect constructions were rated as 0 points.

#### Motor Assessments

We measured participants’ motor abilities with their dominant hand and with a tool while playing a peg-board game (Figure 1). This consists of a series of pegs and a board full of holes where participants had to insert the pegs. In different blocks, participants were tested in three levels of complexity, which differed with respect to the sensorimotor program required: *easy*, *intermediate*, and *difficult*. This depended on the size and shape of the pegs to be used (see Figure 1B and Supplementary Videos).

A single peg is formed by a “foot,” the part that can be inserted into a hole of the platform, and a “head,” the part that can be grasped. The pegs have different head sizes depending on the level of complexity of the task: 15 mm for the *easy* and 5 mm for the *intermediate* and *difficult* level. Independently of the head size, for both *easy* and *intermediate* levels, the pegs have identical foot with a circular section. Participants can therefore place a peg irrespectively of its orientation along the longitudinal axis. However, according to models of sensorimotor control for reaching and grasping (Fitts, 1954; Castiello, 2005) the complexity of the motor manipulation is inverse of the graspable size of the manipulated object. This is also the case for actions performed with a tool as effector (Cardinali et al., 2009). Therefore, the task is expected to be easier when using pegs with 15 mm heads (*easy* level) rather than 5 mm (*intermediate* and *difficult* level).

The *difficult* level, by contrast, added a further sensorimotor constraint: the orientation of the peg along the longitudinal axis. Similarly, to the pegs used in the *easy* and *intermediate* levels, also the pegs employed in the *difficult* one have a direction, that is a “head” (a visible blue stripe on one extremity of the peg) and a “foot.” However, in the *difficult* level the section of the pegs, and of the compatible holes, has a grooved shape. This therefore requires an additional component to be implemented in the grasp-and-place action, corresponding to the appropriate rotation of the peg. Computationally, even the simplest action consists of separate components (e.g., reaching, grip, rotation) which are not independent and need appropriate sequencing (Koechlin and Jubault, 2006; Bongers et al., 2012; Roy et al., 2013). More complex motor tasks are characterized by more complex hierarchies among the different motor sub-units (i.e., grasp – place vs. grasp – rotate – place).

For *easy* and *intermediate* levels, a 22×16 cm plastic board with 27×38 holes for the pegs (Quercetti, “Fantacolor Basic,” product number 2122^1^) was placed in front of the participant at a distance of 45 cm from the edge of the table. For the *difficult* level, two metal platforms with 5×5 grooved holes each (Grooved Pegboard Test, Lafayette instruments, Model 32025^2^) were placed at the same distance on the table, one close to the other (in order to have five rows of 10 holes available).

In each level of complexity, participants were instructed to place 50 pegs in five rows of 10 consecutive elements, as quickly as possible, unaware of a time-limit of 6 min. Participants were instructed to comply with the following rules:

(a) Only one peg per time could be picked up. If accidentally more than one peg were picked up, all had to be dropped back in the box except for one;

(b) The pegs had to be inserted sequentially from left to right without skipping holes, up to complete a row of 10 elements. After the tenth peg, the participant had to start a new line immediately below the leftmost peg of the line just completed;

(c) Participants had to complete five lines. All the holes left erroneously empty counted as incorrect;

(d) If a peg was accidentally dropped, the participant could either pick it up again and keep on with the task or choose to pick a new one;

(e) Only the pegs in the box could be used as new pegs. If a previously dropped peg was collected from the table as a new peg, this counted as incorrect;

(f) A peg had to be fully inserted in the correct hole before moving to pick up the next peg. All the pegs not completely entered in their holes counted as incorrect.

Participants were asked to solve the three levels of complexity once by using their dominant hand (Supplementary Videos 1–3) and once by using the tool. This was a mechanical pinch (30-cm long) and to be acted upon with the right hand (Figure 1A and Supplementary Videos 4–6). To avoid potential order effects, effectors and complexity levels were independently pseudo-randomized across blocks and across participants. At the beginning of each block, the participant could practice on 3 pegs. A 2-min break was given at the end of each block. In each block, an experimenter manually recorded the time to complete each row via a chronometer (Stopwatch, Asaklitt 36-4123) as well as the number of pegs correctly placed. This allowed to index the performance with a motor skill measure expressed by the seconds needed to correctly place a single peg (seconds/peg).

### Statistical Analyses

To assess the reliability between the ratings of two independent judges, we calculated ICC coefficients (McGraw and Wong, 1996), for the Sentence Construction and Repetition scales separately.

To test the selective association between motor performance with the tool and linguistic production abilities, analyses were conducted within the Structural Equation Model (SEM) framework (Kline, 2005; Bollen and Noble, 2011), using AMOS 7.0 (Arbuckle, 2006). To determine whether the effects defined in the model were reliable, differences in χ^2^ fit statistics were used. The alpha level for statistical decisions regarding differences in χ^2^ fit statistics was set to 0.05. Note that a significant chi-square indicates lack of satisfactory model fit.

To test the prediction that the most complex motor task shows the strongest association between motor performance with the tool and linguistic abilities, we run an ANOVA with Complexity as categorical factor (three levels: Easy vs. Intermediate vs. Difficult), and both Sentence Construction and Repetition scores as continuous factors.

## Ethics Statement

All the procedures employed have been approved by the ethics committee (Regionala Etiksprövningsnämnden of Stockholm) and participants gave their written informed consent.

## Author Contributions

CB conceived the study. CB, AR, and ML designed the experiments. CB and LL acquired the data and carried out the analyses. All authors contributed to the final version of the manuscript.

## Conflict of Interest Statement

The authors declare that the research was conducted in the absence of any commercial or financial relationships that could be construed as a potential conflict of interest.

## Footnotes

1 http://www.quercettistore.com/en/products/fantacolor

2 http://www.lafayettelifesciences.com/product_detail.asp?ItemID=167
